# The Johns Hopkins Hospital Reports

**Published:** 1893-09

**Authors:** 


*The Johns Hopkins Hospital Reports.* Vol. III., Nos. 1, 2, 3. *Report in Pathology.* II. Papillomatous Tumours of the Ovary. Tuberculosis of the Female Generative Organs. By J. WHITRIDGE WILLIAMS, M.D. Baltimore: THE JOHNS HOPKINS PRESS. 1892.

We have in this volume much valuable information, collected from all sources, on papillomatous tumours of the ovary and tuberculosis of the female generative organs. English, American, German, and French literature has been extensively used in seeking for this information, and hence we have probably the best papers on these subjects yet published. But it must not be supposed that the report does not contain the results of original work. Dr. Whitridge Williams has been most thoroughly and laboriously investigating the pathology of these conditions. The volume is called a "Report in Pathology"; but it also contains much valuable information with regard to symptoms and treatment, and there are some excellent plates of papillomatous tumours of the ovary.

Dr. Whitridge Williams maintains that the view of English and American pathologists with regard to the origin of these papillomatous cysts—that they arise from remains of the Wolffian body, either in the hilum of the ovary or the parovarium—is not correct. He says English and American pathologists still cling to this theory when most others have forsaken it. Even Olshausen, the originator of the theory, has now totally repudiated it. There seems to be no evidence in favour of it, and these cysts have never been traced microscopically to dilating Wolffian tubes. Recent histological investigations seem to show that these papillomatous cysts are derived from the Graffian follicle, or the germinal epithelium, in the form of ingrowths into the substance of the ovary analogous to Pflüger's ducts, *i.e.* in the same way as multilocular ovarian cysts which do not contain papillomata arise. These tubes could be traced from the surface epithelium into the interior of the ovary, and were seen dilating into cysts lined by columnar and ciliated epithelium, which sometimes contained papillary growths. It was this discovery which induced Olshausen to abandon his theory of origin from remains of the Wolffian body. Dr. Whitridge Williams discovered the microscopic origin of these cysts, both in ingrowths of the surface epithelium and in Graffian follicles. His pictures of the microscopic sections seem to prove his view to be correct.

It is of course now well known that these papillomatous cysts are very liable to rupture, or the papillomatous growth to grow through the wall and infect the peritoneum, so that numerous warty growths spring up over its surface. These grow from minute detached fragments engrafted on the peritoneum, and the term sometimes applied to it of metastasis is inaccurate. There is nothing malignant about it: it happens simply because very small particles can be easily detached, and will continue to grow when grafted on the peritoneum; and the result of operation on these cases when the growth has become disseminated is usually quite satisfactory, as in a case which occurred at the Bristol General Hospital, reported in the *Lancet* of last year. But Dr. Williams refers to a few cases in which there have been true metastases. In one there were several subcutaneous papillomatous cysts; in another, papillomatous growths (evidently of embolic origin) in the large blood-vessels; in a third, papillomatous cysts in the pleura; and in a fourth, similar growths in the lymphatic glands behind the peritoneum and above the diaphragm. Some of these secondary growths in the pleura were probably started by emboli infecting the throracic lymph glands through the lymphatics of the diaphragm, and these in turn infecting the pleura.

Dr. Williams also points out that the papillomatous cysts are particularly liable to become cancerous, in a strictly histological sense, and at the same time clinically malignant. We are surprised to read that this frequently occurs—as often as 40 per cent. of the cases operated on by Schroeder. It has not, we believe, been recognised in this country, and as papillomatous cysts often occur fairly early in life, such a percentage is astonishing.

Superficial papillomata of the ovary are also treated of in this volume. Williams has traced their growth from the germinal (surface) epithelium. Olshausen, writing in 1886, stated that he had only been able to find six cases recorded. As Williams came across five cases in a comparatively short period, he thought they could not be quite so rare, and he searched and found seven more. But we think very great care is necessary in the examination of these cases before we can conclude that the growth was not originally contained in a cyst. Williams refers to two specimens in the museum of the College of Surgeons in London, the reference to which he discovered in the museum catalogue; but with regard to both specimens, it is stated that the growths were probably at an early stage intra-cystic. Often, only some torn shreds of the cyst wall remain.

And now we come to the second subject treated of in this volume—tuberculosis of the female generative organs. In 1888 Dr. W. S. A. Griffiths, Assistant Obstetric Physician to St. Bartholomew's Hospital, brought before the Pathological Society of London a specimen of tubercle of the ovary, and he then collected all the literature on the subject which he could find. He discovered twenty-four papers on the subject—only five in the English language. He says: "Our museums, with the exception of St. George's, St. Thomas', and Guy's, so far as I have been able to ascertain, contain no specimens." Williams has referred to 133 papers on the subject, chiefly German and French, and hence the great value of his communication. He has also a report of nine cases he has himself examined; but, unfortunately, space will not allow us to refer in detail to his interesting work.

